# Supplementary material for: Landscape fragmentation and pollinator movement within agricultural environments: a modelling framework for exploring foraging and movement ecology
Source: PeerJ. 2014 Feb 27;2:e269. doi: 10.7717/peerj.269 (PMC3940622; doi:10.7717/peerj.269)
Supplement: Supplemental Information 10 [file peerj-02-269-s010.pdf]

SUPPLEMENTARY INFORMATION

TABLES OF POST-HOC TEST RESULTS

All tables report *Z* values for Tukey tests, with: '*' $p < 0.05$, '**' $p < 0.005$, '***' $p < 0.001$

MODEL 1: EFFECTS OF MOVEMENT CHOICE
Pairwise comparisons of probability of moving forwards

maximum distance from nest

	0.25	0.375	0.5	0.625	0.75
0.375	*3.077				
0.5	***12.822	***9.745			
0.625	***22.381	***19.304	***9.560		
0.75	***34.218	***31.141	***21.396	***11.836	
0.875	***74.132	***71.055	***61.31	***51.751	***39.914

number of habitat changes

	0.25	0.375	0.5	0.625	0.75
0.375	1.313				
0.5	-0.103	-1.415			
0.625	2.552	1.239	2.654		
0.75	-0.426	-1.739	-0.323	*-2.978	
0.875	1.759	0.447	1.892	-0.792	2.185

proportion of time spent in wild

	0.25	0.375	0.5	0.625	0.75
0.375	-1.239				
0.5	*-3.109	-1.87			
0.625	-2.497	-1.258	0.612		
0.75	***-6.347	***-5.108	*-3.238	**-3.850	
0.875	***-7.894	***-6.655	***-4.785	***-5.397	-1.547

MODEL 2: EFFECTS OF ALTERING THE LIKELIHOOD OF SWITCHING PREFERENCE
Pairwise comparisons of probability of switching

maximum distance from nest

	0.00513823	0.00770735	0.011561	0.0173415	0.0260123	0.0390184	0.0585277	0.0877915	0.131687	0.197531	0.296296	0.444444	0.666667
0.00770735	3.076												
0.011561	***4.955	1.879											
0.0173415	***4.949	1.873	-0.006										
0.0260123	***7.361	**4.285	2.406	2.412									
0.0390184	***6.992	**3.916	2.038	2.044	-0.368								
0.0585277	***8.869	***5.793	**3.914	**3.92	1.508	1.876							
0.0877915	***9.298	***6.222	**4.343	**4.349	1.937	2.305	0.429						
0.131687	***9.096	***6.019	**4.141	**4.147	1.735	2.103	0.227	-0.202					
0.197531	***12.57	***9.494	***7.615	***7.621	***5.209	***5.577	*3.701	3.272	*3.474				
0.296296	***12.878	***9.802	***7.923	***7.93	***5.518	***5.886	**4.01	*3.580	*3.783	0.308			
0.444444	***12.844	***9.768	***7.889	***7.895	***5.483	***5.851	**3.975	*3.546	*3.748	0.274	-0.034		
0.666667	***16.013	***12.937	***11.059	***11.065	***8.653	***9.021	***7.145	***6.716	***6.918	*3.443	3.135	3.170	
1	***14.44	***11.364	***9.486	***9.492	***7.08	***7.448	***5.572	***5.143	***5.345	1.87	1.562	1.596	-1.573

number of habitat changes

	0.00513823	0.00770735	0.011561	0.0173415	0.0260123	0.0390184	0.0585277	0.0877915	0.131687	0.197531	0.296296	0.444444	0.666667
0.00770735	1.344												
0.011561	2.824	1.480											
0.0173415	***4.692	3.348	1.868										
0.0260123	***7.373	***6.030	***4.550	2.681									
0.0390184	***10.752	***9.409	***7.929	***6.060	*3.379								
0.0585277	***14.712	***13.368	***11.888	***10.020	***7.338	**3.959							
0.0877915	***20.087	***18.743	***17.263	***15.395	***12.713	***9.334	***5.375						
0.131687	***26.108	***24.764	***23.284	***21.416	***18.735	***15.356	***11.396	***6.021					
0.197531	***33.141	***31.797	***30.317	***28.449	***25.767	***22.388	***18.429	***13.054	***7.033				
0.296296	***41.010	***39.666	***38.186	***36.318	***33.636	***30.257	***26.298	***20.923	***14.901	***7.869			
0.444444	***49.159	***47.815	***46.335	***44.467	***41.786	***38.407	***34.447	***29.072	***23.051	***16.018	***8.149		
0.666667	***64.647	***63.303	***61.823	***59.955	***57.273	***53.894	***49.935	***44.560	***38.539	***31.506	***23.637	***15.488	
1	***77.903	***76.559	***75.079	***73.211	***70.530	***67.151	***63.191	***57.816	***51.795	***44.762	***36.893	***28.744	***13.256

proportion of time spent in wild

	0.00513823	0.00770735	0.011561	0.0173415	0.0260123	0.0390184	0.0585277	0.0877915	0.131687	0.197531	0.296296	0.444444	0.666667
0.00770735	***-27.188												
0.011561	***-27.021	0.167											
0.0173415	***-41.304	***-14.116	***-14.283										
0.0260123	***-44.825	***-17.637	***-17.804	*-3.521									
0.0390184	***-44.412	***-17.224	***-17.391	-3.108	0.413								
0.0585277	***-46.54	***-19.352	***-19.518	***-5.235	-1.715	-2.128							
0.0877915	***-46.736	***-19.548	***-19.715	***-5.432	-1.911	-2.324	-0.196						
0.131687	***-47.876	***-20.688	***-20.855	***-6.572	-3.051	*-3.464	-1.337	-1.140					
0.197531	***-48.370	***-21.182	***-21.348	***-7.065	*-3.545	**-3.958	-1.830	-1.634	-0.493				
0.296296	***-48.553	***-21.366	***-21.532	***-7.249	*-3.728	**-4.141	-2.014	-1.817	-0.677	-0.184			
0.444444	***-49.121	***-21.933	***-22.099	***-7.817	**-4.296	***-4.709	-2.581	-2.385	-1.245	-0.751	-0.567		
0.666667	***-48.476	***-21.288	***-21.455	***-7.172	*-3.651	**-4.064	-1.936	-1.740	-0.600	-0.106	0.077	0.645	
1	***-48.101	***-20.913	***-21.080	***-6.797	-3.276	*-3.689	-1.561	-1.365	-0.225	0.269	0.453	1.020	0.375

MODEL 3: EFFECTS OF INCLUDING SET-ASIDES
Pairwise comparisons of number of set-asides

maximum distance from nest

	0	5	10	15	20	25	30	35	40	45
5	***5.259									
10	***5.279	0.020								
15	***5.786	0.527	0.507							
20	***5.630	0.370	0.351	-0.156						
25	***6.242	0.983	0.963	0.457	0.613					
30	***6.713	1.454	1.434	0.927	1.083	0.470				
35	***6.805	1.545	1.526	1.019	1.175	0.562	0.092			
40	***6.483	1.224	1.204	0.697	0.853	0.241	-0.230	-0.322		
45	***6.225	0.966	0.946	0.439	0.596	-0.017	-0.488	-0.579	-0.258	
50	***5.898	0.639	0.619	0.112	0.269	-0.344	-0.815	-0.906	-0.585	-0.327

number of habitat changes

	0	5	10	15	20	25	30	35	40	45
5	-1.718									
10	**-4.028	-2.31								
15	***-7.149	***-5.431	-3.121							
20	***-9.222	***-7.504	***-5.194	-2.073						
25	***-12.160	***-10.442	***-8.132	***-5.012	-2.938					
30	***-15.116	***-13.398	***-11.088	***-7.967	***-5.894	-2.956				
35	***-17.290	***-15.572	***-13.262	***-10.141	***-8.068	***-5.130	-2.174			
40	***-19.048	***-17.330	***-15.02	***-11.899	***-9.826	***-6.887	**-3.932	-1.758		
45	***-21.207	***-19.489	***-17.179	***-14.058	***-11.985	***-9.047	***-6.091	**-3.917	-2.159	
50	***-23.596	***-21.878	***-19.568	***-16.447	***-14.374	***-11.436	***-8.480	***-6.306	***-4.548	-2.389

proportion of time spent in wild

	0	5	10	15	20	25	30	35	40	45
5	***5.340									
10	***8.874	*3.534								
15	***13.093	***7.753	**4.218							
20	***17.149	***11.808	***8.274	**4.056						
25	***21.430	***16.090	***12.556	***8.337	***4.281					
30	***26.322	***20.982	***17.448	***13.229	***9.173	***4.892				
35	***30.667	***25.327	***21.793	***17.575	***13.519	***9.237	***4.345			
40	***33.973	***28.633	***25.099	***20.880	***16.824	***12.543	***7.651	*3.306		
45	***36.792	***31.452	***27.918	***23.699	***19.643	***15.362	***10.470	***6.125	*2.819	
50	***41.079	***35.738	***32.204	***27.986	***23.930	***19.648	***14.757	***10.411	***7.106	***4.287

MODEL 3a: EFFECTS OF INCLUDING SET-ASIDES WHEN EES DO NOT CHANGE HABITATS
Pairwise comparisons of number of set-asides

maximum distance from nest

	0	5	10	15	20	25	30	35	40	45
5	2.198									
10	***5.156	2.958								
15	***7.728	***5.530	2.572							
20	***10.214	***8.017	***5.059	2.486						
25	***12.164	***9.967	***7.008	***4.436	1.950					
30	***15.346	***13.148	***10.19	***7.618	***5.132	3.182				
35	***16.760	***14.562	***11.604	***9.032	***6.546	***4.596	1.414			
40	***19.233	***17.035	***14.077	***11.505	***9.018	***7.069	***3.887	2.473		
45	***21.301	***19.104	***16.145	***13.573	***11.087	***9.137	***5.955	***4.541	2.068	
50	***22.699	***20.501	***17.543	***14.971	***12.484	***10.534	***7.353	***5.939	*3.466	1.397

MODEL4: EFFECTS OF REMOVING HEDGES
Pairwise comparisons of number of hedges removed

maximum distance from nest

	0	4	8	12	16	20	24	28	32	36
4	**3.913									
8	***5.785	1.872								
12	1.389	-2.524	***-4.396							
16	*3.425	-0.487	-2.360	2.036						
20	*3.531	-0.381	-2.254	2.142	0.106					
24	3.051	-0.862	-2.734	1.662	-0.374	-0.480				
28	2.081	-1.832	**-3.704	0.692	-1.345	-1.451	-0.970			
32	1.359	-2.554	***-4.426	-0.030	-2.066	-2.172	-1.692	-0.722		
36	***4.825	0.912	-0.960	*3.436	1.400	1.294	1.774	2.744	*3.466	
40	**3.886	-0.027	-1.899	2.496	0.460	0.354	0.835	1.805	2.526	-0.940

number of habitat changes

	0	4	8	12	16	20	24	28	32	36
4	-2.067									
8	-1.08	0.987								
12	-3.094	-1.027	-2.015							
16	-2.466	-0.399	-1.386	0.628						
20	-2.710	-0.643	-1.630	0.384	-0.244					
24	***-4.601	-2.534	*-3.521	-1.507	-2.135	-1.891				
28	*-3.603	-1.537	-2.524	-0.509	-1.138	-0.893	0.998			
32	***-5.741	*-3.675	***-4.662	-2.647	*-3.276	-3.032	-1.141	-2.138		
36	***-5.782	**-3.715	***-4.702	-2.688	*-3.316	-3.072	-1.181	-2.178	-0.040	
40	***-6.953	***-4.887	***-5.874	**-3.859	***-4.488	**-4.244	-2.353	*-3.350	-1.212	-1.172

proportion of time spent in wild

	0	4	8	12	16	20	24	28	32	36
4	-0.938									
8	-0.924	0.013								
12	-2.809	-1.872	-1.885							
16	-2.02	-1.082	-1.096	0.789						
20	-2.857	-1.919	-1.932	-0.047	-0.837					
24	-3.131	-2.193	-2.206	-0.321	-1.111	-0.274				
28	-2.668	-1.73	-1.744	0.141	-0.648	0.189	0.462			
32	***-4.659	**-3.722	**-3.735	-1.850	-2.639	-1.803	-1.529	-1.991		
36	*-3.640	-2.702	-2.715	-0.830	-1.620	-0.783	-0.509	-0.972	1.020	
40	***-5.416	***-4.478	***-4.491	-2.606	*-3.396	-2.559	-2.285	-2.748	-0.756	-1.776

MODEL4a: EFFECTS OF REMOVING HEDGES WHEN BEES DO NOT CHANGE HABITAT
Pairwise comparisons of number of hedges removed

maximum distance from nest

	0	4	8	12	16	20	24	28	32	36
4	1.086									
8	1.687	0.601								
12	0.575	-0.512	-1.112							
16	-0.072	-1.159	-1.759	-0.647						
20	1.757	0.671	0.070	1.182	1.829					
24	1.672	0.585	-0.015	1.097	1.744	-0.085				
28	2.595	1.509	0.908	2.021	2.668	0.838	0.924			
32	1.736	0.650	0.049	1.162	1.809	-0.021	0.065	-0.859		
36	2.932	1.846	1.245	2.358	3.005	1.175	1.261	0.337	1.196	
40	2.885	1.798	1.198	2.310	2.957	1.128	1.213	0.289	1.148	-0.048
